# The prognostic value of postoperative platelet levels in elderly patients after valve replacement surgery: a retrospective cohort study

**DOI:** 10.1186/s12872-024-04041-4

**Published:** 2024-07-22

**Authors:** Yuxin Nong, Xuebiao Wei, Junquan Lu, Danqing Yu

**Affiliations:** 1 Department of Cardiology, Guangdong Provincial Key Laboratory of Coronary Heart Disease Prevention, Guangdong Cardiovascular Institute, Guangdong Provincial People’s Hospital, Guangdong Academy of Medical Sciences, Guangzhou, 510080 China; 2 Department of Geriatric Intensive Medicine, Guangdong Provincial Geriatrics Institute, Guangdong Provincial People’s Hospital, Guangdong Academy of Medical Sciences, Guangzhou, 510080 China

**Keywords:** Postoperative platelet levels, Elderly, Valve replacement, Prognosis

## Abstract

**Background:**

Further research is needed to assess the risk and prognosis after valve replacement surgery in elderly patients. This study aims to assess the prognostic value of platelet levels following valve replacement in elderly patients.

**Methods:**

A retrospective analysis was conducted on 3814 elderly individuals who underwent valve replacement surgery, categorized into quartiles based on postoperative platelet levels. Univariate and multiple regression analysis were used to assess the risk factors associated with postoperative platelet levels and in-hospital death.The Receiver Operating Characteristic (ROC) curve was utilized to establish the postoperative platelet level threshold indicative of in-hospital mortality risk, while the Kaplan-Meier curve compared the one-year postoperative survival among patients with differing postoperative platelet levels.

**Results:**

The low postoperative platelet levels group had a higher incidence of massive bleeding (> 400 ml), necessitating platelet transfusion and prolonged cardiopulmonary bypass during surgery (*P* < 0.001). However, postoperative occurrences of heart failure and stroke did not achieve statistical significance (*P* > 0.05). Multivariate regression analysis disclosed an association between postoperative platelet levels and in-hospital death (OR: 2.040, 95% CI: 1.372–3.034, *P* < 0.001). Over the one-year follow-up, patients with low platelet levels postoperatively had poorer overall survival than patients with higher platelet levels (*P* < 0.001)

**Conclusion:**

Postoperative platelets can serve as a prognostic indicator after valve surgery in elderly patients as a simple and easily available biochemical indicator. Enhanced monitoring and management postoperative platelet level in the elderly may be beneficial to improve the survival outcome of patients

## Introduction

Valvular heart disease (VHD) ranks among the most prevalent cardiovascular disorders, afflicting approximately 30 million individuals worldwide, with an annual mortality toll of 130,000. As societies continue to age at an accelerated pace, it is anticipated that these numbers will further ascend [1]. Currently, the main measure of radical treatment for VHD is valve replacement surgery. The association between age and the susceptibility to valve disease becomes more pronounced in our current era of demographic aging. For elderly patients with no contraindications, the quality of life can be greatly improved through valve replacement surgery [2]. A study based on a large national database showed that for elderly patients without severe complications, valve surgery can help to improve survival and enhance their quality of life [3]. Concurrently, advancements in minimally invasive valve surgery also led to a reduction in the hospitalization duration for elderly patients undergoing valve replacement surgery [4].

However, there is no denying that as the physical functions of older adults decline, there may be an increased risk of perioperative complications [5]. Despite numerous studies highlighting the substantial benefits of valve replacement in terms of survival and symptom relief, the areas of preoperative assessment and perioperative management remain subjects of ongoing discourse. A multicenter prospective cohort study showed that frailty, including lower limb weakness, cognitive impairment, anemia, and hypoalbuminemia indicators, were strong determinants of death and disability within 1 year of valve surgery in elderly patients [6]. In conclusion, while advanced age should not serve as grounds for surgical refusal, the optimization of perioperative care and the early identification of high-risk factors for postoperative adverse events are pressing concerns.

Studies have shown that patients with low postoperative platelet levels after liver surgery have a poor prognosis, which may be caused by the stress and depletion of platelets during surgery [7]. Moreover, postoperative thrombocytopenia has also been reported to be associated with prolonged mechanical ventilation, increased tendency to hemorrhage and hospital stay [8]. Nevertheless, the impact of postoperative platelet levels on the prognosis of elderly patients undergoing valve surgery remains unclear. Postoperative platelet levels after valve surgery may significantly influence patients, particularly due to the involvement of cardiopulmonary bypass (CPB), stricter postoperative anticoagulant management, and the heightened thrombosis risk in older individuals. In this study, we performed a retrospective analysis to evaluate the prognostic value of platelet levels after valve surgery in elderly patients.

## Methods

### Sample source and study design

This retrospective observational study focused on patients aged 60 and above who underwent valve replacement surgery at Guangdong Provincial People’s Hospital from 2010 to 2017. The following conditions will be excluded: (1) no postoperative platelet records (2) infectious diseases, immune disorders, such as hepatitis (3) malignant tumors (4) intraoperative death. The prevalent valve diseases included aortic stenosis, aortic regurgitation, mitral stenosis, mitral regurgitation, and tricuspid regurgitation. Aortic valve replacement (29.6%), mitral valve replacement (34.4%), and tricuspid valve replacement (30.0%) constituted the most frequent surgical interventions. Surgery and perioperative care of patients was performed by the heart team at the hospital according to current practice guidelines. Warfarin was administered for at least 3 to 6 months and coagulation status was monitored regularly after surgery to maintain the international normalized ratio between 1.8 and 2.5. Then, Aspirin (100 mg/d) or clopidogrel (75 mg/d) was then administered for long-term maintenance [9]. Patients were categorized into four groups based on platelet levels determined within 12 h post-surgery using the first intravenous test. Blood collection was undertaken by a professional nurse in the surgical intensive care unit and analyzed utilizing an automated counter instrument (Beckman Coulter, Brea, CA). The study was conducted in accordance with the Declaration of Helsinki (as revised in 2013). The study was approved by institutional ethics board of Guangdong Provincial People’s Hospital (NO: GDREC2018525H).

### Data collection

Patient covariates included fundamental clinical history and postoperative laboratory data, including gender, age, history of hypertension, history of diabetes, prior valve surgery, New York Heart Association cardiac function classification (NYHA) at admission, left ventricular ejection fraction (LVEF) at admission, creatinine level at admission, preoperative white blood cell level (WBC), anemia status, alanine aminotransferase (ALT) level, estimated glomerular filtration rate (eGFR), platelet level, type of aortic valve disease, type of surgery, whether concomitant coronary artery bypass grafting was performed, duration of extracorporeal circulation, intraoperative blood loss, and platelet transfusion during surgery.

### Patient outcome and follow-up

The primary outcomes for patients included in-hospital mortality and survival within the initial year following surgery. The postoperative complications such as re-thoracotomy for hemostasis, dialysis, acute heart failure and stroke was compared between different postoperative platelets group. Postoperative survival duration was defined as the period from the surgical intervention to either the occurrence of mortality or the lapse of 1 year. Patients were followed up facilitated by skilled nursing professionals through methods such as telephone communication, in-person outpatient interviews, and scrutiny of outpatient medical records.

### Statistics and analysis

Continuous variables were presented as mean + standard deviation or interquartile range (IQR) and compared by using One-Way test or Kruskal-Wallis test. Categorical variables were expressed as counts and percentages, and comparisons were conducted by using chi-square tests. The prognostic variables were evaluated by univariate logistic regression and adjusted by multivariate analysis. The receiver operating characteristic (ROC) curve was used to determine the optimal cut-off value for platelet prediction of in-hospital death, and the area under the curve (AUC) was calculated to evaluate the accuracy of the prediction. The Kaplan-Meier curve was used to express the survival outcome of patients and tested by log-rank. The above statistics were calculated by R 4.2 (https://www.r-project.org/), and two-sided *P* < 0.05 was considered statistically significant.

## Results

### Clinical baseline characteristics

There were 3814 patients enrolled, including 1994 males (52.3%) and 1820 females (47.7%). Patients were divided into four groups based on the postoperative platelet levels quartiles: Q1:<94 × 10^9/L, Q2: (94–119)×10^9/L, Q3:(119–149)×10^9/L, Q4: ≥149 × 10^9/L. The distribution of patients across these quartiles was as follows: Q1: 952(25.0%), Q2: 941(24.7%), Q3: 960(25.2%), Q4: 961(25.2%). Subsequent to surgery, patients with low platelet levels had a higher incidence of bleeding (> 400 ml) (*P* < 0.001), more platelet transfusion (*P* < 0.001), and a longer period of CPB (*P* < 0.001). The baseline characteristics of the patients are shown in Table 1.

**Table 1:** Clinical baseline characteristics of the patients

	Q1	Q2	Q3	Q4	*p* value
n	952	941	960	961	
Age	65.5 (4.2)	65.0 (4.1)	65.3 (4.5)	65.1 (4.2)	0.047
Gender(female) n (%)	413 (43.4)	433 (46.0)	500 (52.1)	474 (49.3)	< 0.001
Hypertension n (%)	265 (27.8)	282 (30.0)	355 (37.0)	362 (37.7)	< 0.001
Diabetes n (%)	104 (10.9)	104 (11.1)	123 (12.8)	123 (12.8)	0.39
Previous valve replacement n (%)	29 (3.0)	34 (3.6)	39 (4.1)	43 (4.5)	0.40
NYHA (III-IV)	378 (39.7)	309 (32.8)	310 (32.3)	351 (36.5)	0.002
Preoperative measurement					
Scr (umol/L)	90.5 (34.5)	85.6 (26.3)	85.1 (28.9)	86.6 (33.2)	< 0.001
ALT (U/L)	20.0[15.8, 28.0]	20.0[15.0, 27.0]	19.0[15.0, 27.0]	19.0[14.5, 27.0]	0.24
WBC count (10^9 /L)	6.6 (2.1)	6.6 (1.9)	6.9 (2.2)	7.6 (2.3)	< 0.001
Hemoglobin (g/L)	132.1 (16.8)	131.2 (16.8)	129.5 (16.5)	126.6 (18.2)	< 0.001
Platelet count (10^9 /L)	151.1 (45.5)	173.3 (43.0)	195.9 (49.9)	234.1 (68.6)	< 0.001
Severe valve disease n (%)					
Aortic stenosis	252 (26.5)	191 (20.3)	198 (20.6)	188 (19.6)	< 0.001
Aortic regurgitation	203 (21.3)	213 (22.6)	227 (23.6)	243 (25.3)	0.21
Mitral stenosis	214 (22.5)	201 (21.4)	174 (18.1)	154 (16.0)	< 0.001
Mitral regurgitation	383 (40.2)	387 (41.1)	382 (39.8)	399 (41.5)	0.86
Tricuspid regurgitation	245 (25.7)	213 (22.6)	216 (22.5)	196 (20.4)	0.049
LVEF n (%)	60.9 (10.8)	61.0 (9.9)	61.2 (10.1)	60.8 (10.3)	0.9
Type of surgery n (%)					
AVR	562 (59.0)	566 (60.1)	559 (58.2)	558 (58.1)	0.785
MVR	658 (69.1)	653 (69.4)	653 (68.0)	594 (61.8)	0.001
TVI	593 (62.3)	571 (60.7)	549 (57.2)	491 (51.1)	< 0.001
Concomitant CABG	122 (12.8)	98 (10.4)	118 (12.3)	108 (11.2)	0.365
CPB duration (min)	153.6 (66.8)	144.6 (57.5)	134.4 (53.0)	136.0 (51.8)	< 0.001
Intraoperative n (%)					
Blood loss (> 400mL)	393 (41.8)	302 (32.4)	277 (29.1)	252 (26.8)	< 0.001
Platelet transfusion	153 (16.3)	193 (20.7)	215 (22.6)	297 (31.5)	< 0.001
Postoperative platelet count	75.0[66.2,87.4]	106.2[100.8,112.0]	133.2[125.6,140.3],	184.6[158.8,197.1]	< 0.001

Scr: serum creatinine; ALT: alanine aminotransferase; WBC: white blood cell level; LVEF: left ventricular ejection fraction; AVR: aortic valve replacement; MVR: mitral valve replacement; TVR: tricuspid valve replacement; CABG: coronary artery bypass grafting; CPB: cardiopulmonary bypass duration

### Postoperative platelet levels and postoperative complications

**Fig. 1:**
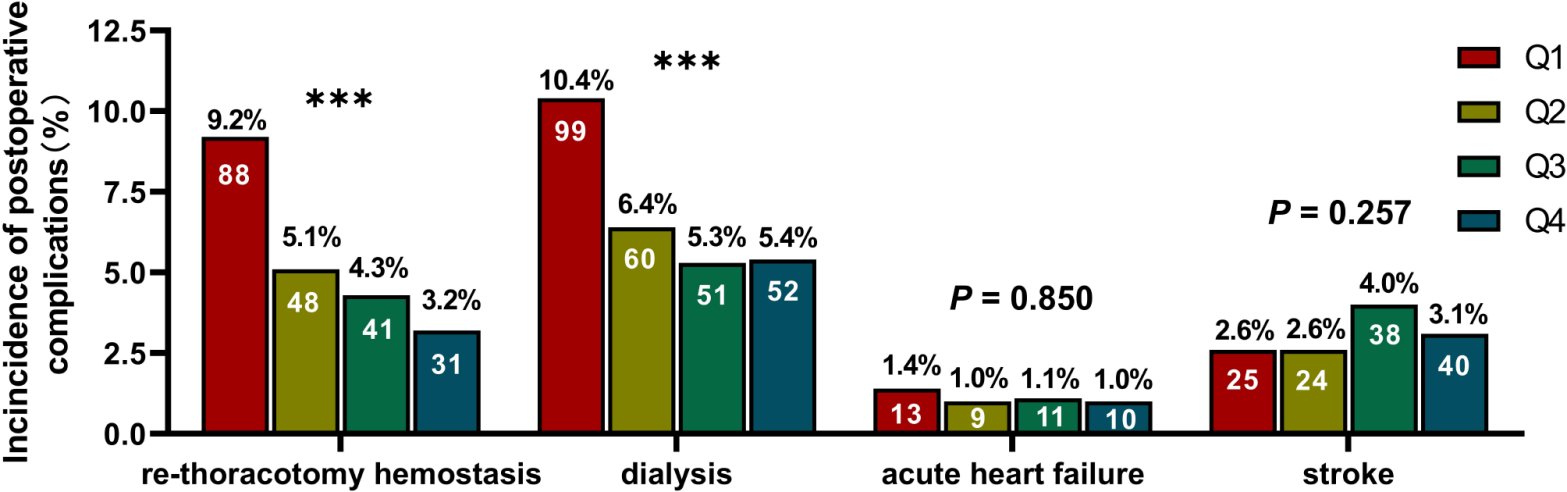
The incidence of complications in patients with different postoperative platelet levels

We found that patients with low platelet levels after surgery had a higher rate of re-thoracotomy for hemostasis and dialysis after surgery (*P* < 0.001). However, we found no statistical significance in postoperative acute heart failure and stroke (*P* < 0.05), as shown in Fig. 1.

### Association postoperative between platelet level and in-hospital death

**Fig. 2:**
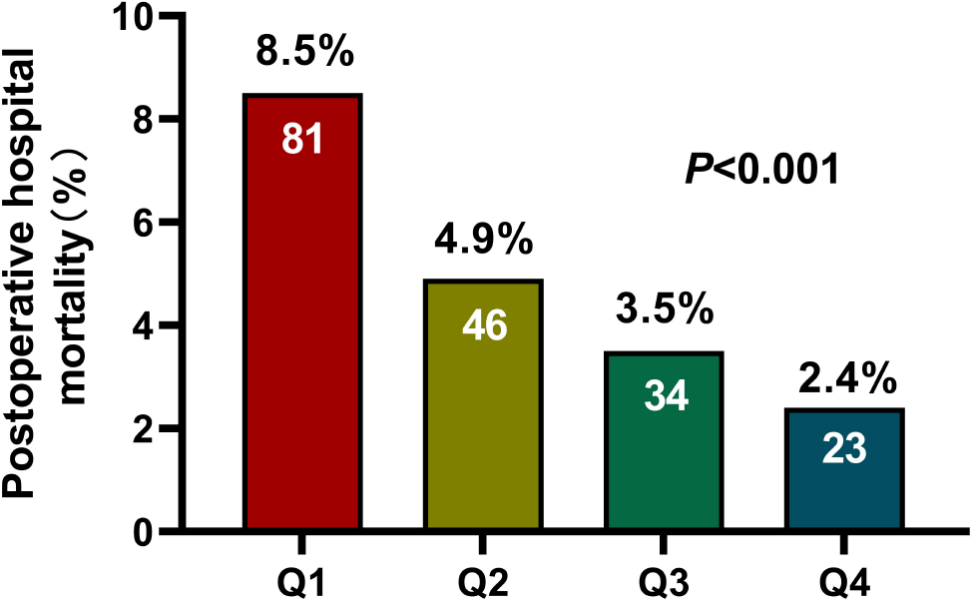
The hospital mortality in patients with different postoperative platelet levels

**Fig. 3:**
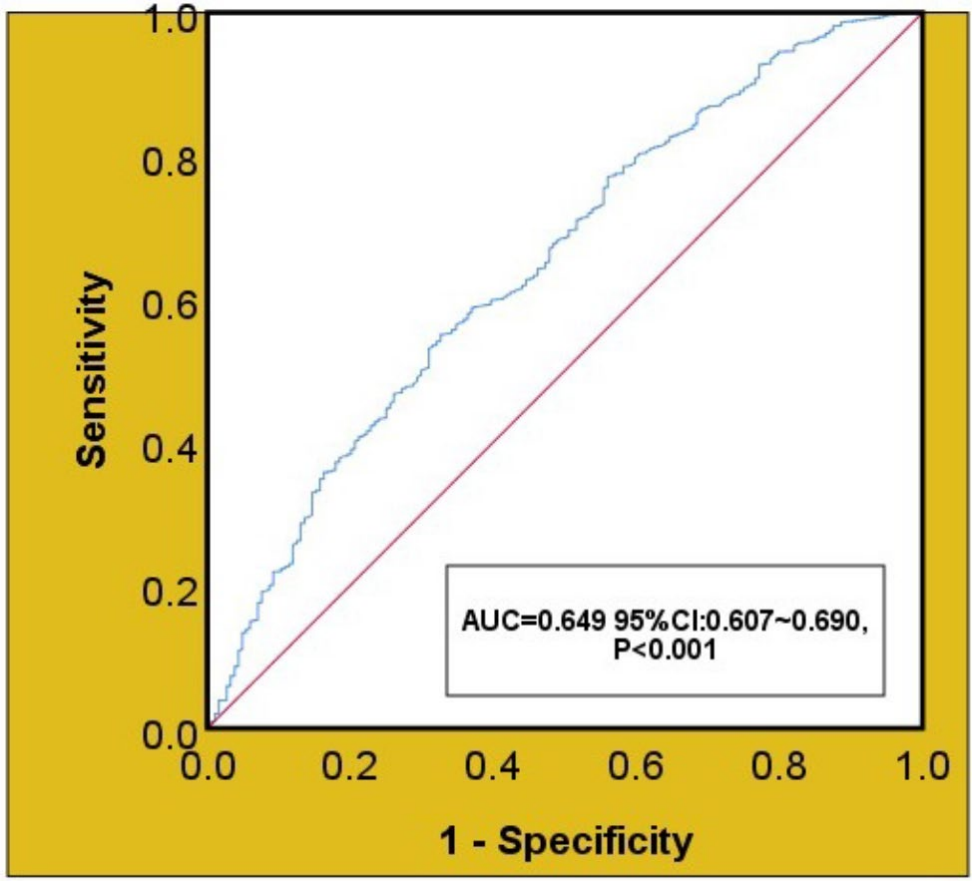
Receiver operating characteristic curve of in-hospital deaths after surgery

**Fig. 4:**
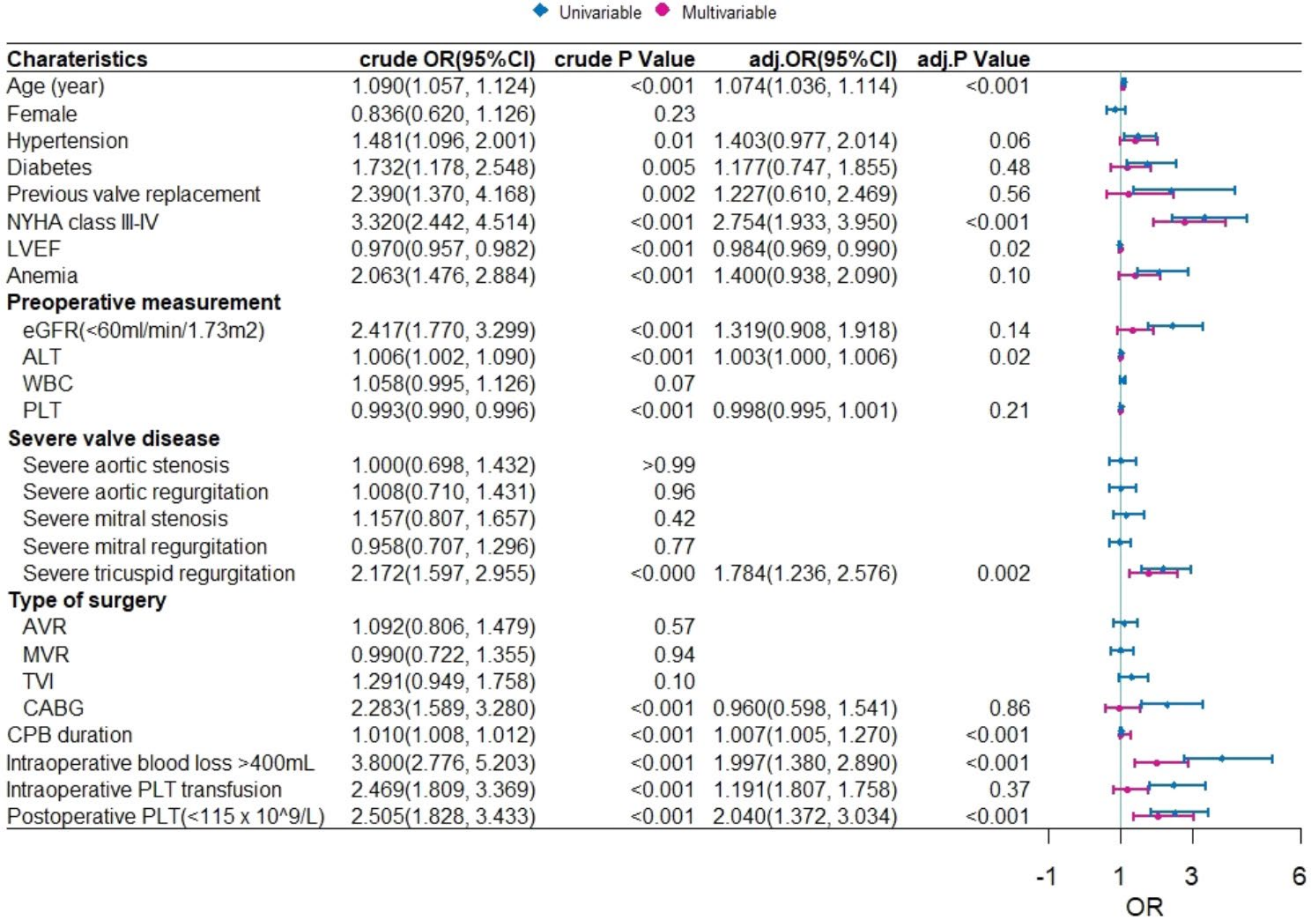
Univariate and multivariate regression analyses of in-hospital deaths

Our results showed that patients with lower postoperative platelets had a higher rate of in-hospital mortality (*P* < 0.001), as shown in Fig. 2. The results of univariate regression analysis showed that hospital deaths were associated with age, female, hypertension, diabetes, previous valve replacement, NYHA, eGFR, ALT, anemia, severe valve disease, and type of surgery. The ROC curve analysis showed that the optimal platelet prediction node was 115 × 10^9^/L, which had a sensitivity of 55% and a specificity of 67.4% (AUC = 0.649, 95% CI: 0.607–0.690, *P* < 0.001), as shown in Fig. 3. After multiple regression adjustment, postoperative platelet level was found to be an independent prognostic factor (OR: 2.040, 95% CI: 1.372–3.034, *P* < 0.001), as shown in Fig. 4.

### Prognosis of different platelet levels

**Fig. 5:**
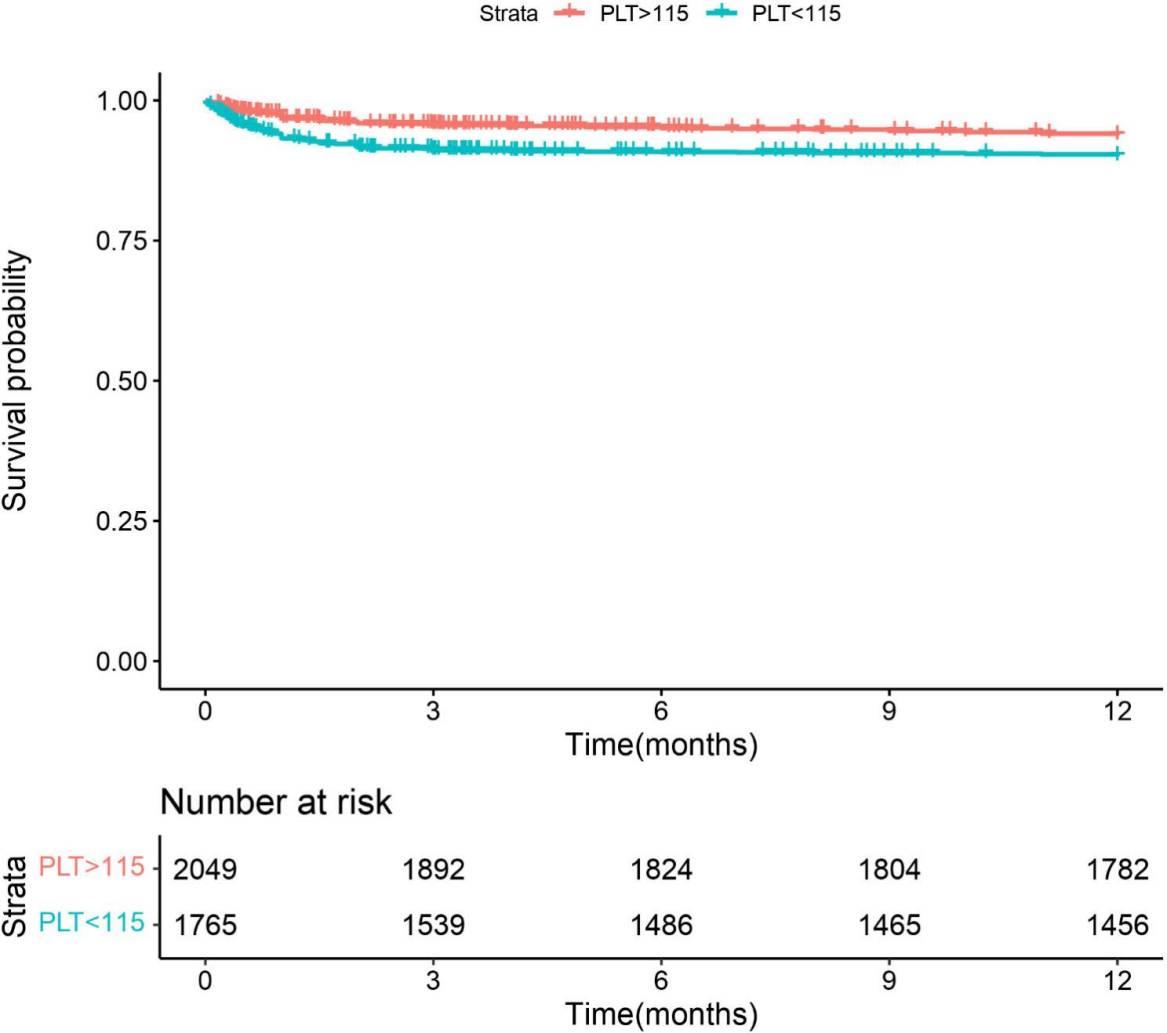
1-year survival curves of patients with different postoperative platelet levels

After 1 year of follow-up, we found that the overall survival of patients with postoperative low platelet (< 115 × 10^9/L) was significantly lower than that of patients with high platelet level (log rank = 0.868, *P* < 0.001), as shown in Fig. 5.

## Discussion

This study explored the correlation between postoperative platelet levels and patient outcomes in a cohort of 3,814 individuals. To the best of our knowledge, this is the first study to examine postoperative platelet levels and the outcome of valve replacement surgery in the elderly in a large sample. Our study found that the lower the postoperative platelet levels of patients, the higher the proportion of patients requiring re-thoracotomy hemostasis and dialysis. On the one hand, this may be due to platelet depletion caused by bleeding and drug application during the surgery, which leads to decreased coagulation function [10]. On the other hand, activated platelets release bioactive granules, including alpha granules and dense granules, fostering interactions with immune cells in the bloodstream and organs [11, 12]. Aberrantly activated platelets might potentially adhere to and accumulate in the vascular bed of renal tissue, augmenting leukocyte recruitment and influencing kidney function [13]. These complications may further increase the risk of in-hospital death.

In previous studies, stroke has been found to be a common complication of valve surgery [14]. However, our study did not establish a discernible association between postoperative platelet levels and acute heart failure or stroke. This disparity could be attributed to the limited number of recorded outcomes, and the potential underreporting of minor stroke events by patients post-discharge. In certain instances of stroke and acute heart failure, out-of-hospital deaths may also go unreported.

Furthermore, our investigation identified postoperative platelet levels as an independent risk factor for in-hospital mortality. According to the ROC curve, the critical threshold for the postoperative platelet level associated with hospital mortality risk was determined to be 115 × 10^9/L, demonstrating predictive value for survival outcomes one-year post-surgery. Individuals with coagulation disorders face an elevated likelihood of encountering adverse events post-surgery, thereby diminishing their prospects of survival [15]. This aligns with previous studies demonstrating the robust predictive capability of platelet count in the context of sepsis-related mortality within specialized care units [16].

Platelets play a pivotal role in hemostasis in the human body. There is growing evidence that platelets play an important role in the occurrence and progression of diseases, and they have been used as new predictors in disease risk assessment [17]. Notably, platelet activation induced by CPB during cardiac surgery has been linked to an elevated risk of postoperative thrombocytopenia [18, 19]. Given the indispensability of anticoagulant measures following valve replacement, the use of anticoagulant medications may heighten the risk of adverse events, particularly bleeding in the presence of low platelet counts [20]. This risk is further exacerbated when drugs like heparin are employed during surgery, contributing to postoperative thrombocytopenia [21, 22]. It is well-established that platelets exhibit significant variations in number, functional activity, and structure between older and younger individuals, so platelets in the elderly appear to be more susceptible to activation, thereby predisposing them to thrombotic events [23]. Consequently, optimizing platelet management during the perioperative period holds promise for enhancing both the survival and prognostic outcomes of elderly patients.

There are several limitations to our study. Firstly, it is essential to note that our investigation is a single-center retrospective study, potentially introducing patient selection bias. Secondly, We used and compared patients’ postoperative static platelet levels rather than dynamic levels, so we could not detect changes in platelet count trajectories in patients, which may have caused us to lose part of the information. A more detailed level curve may be more conducive to real-time assessment and prediction of patient risk status. Thus, We look forward to further establishing a postoperative prognostic warning model for patients by establishing a unified and standardized platelet dynamic trajectory. Then, despite our efforts to account for potential confounding variables, there remains the possibility of other unmeasured confounders, encompassing variations in surgical procedures, postoperative care, social support following discharge, and potential complications. Finally, the majority of patients who undergo valve replacement surgery are 65, which may be considered relatively “young” and may not fully represent the elderly population in developed countries. Therefore, we anticipate multi-center studies with larger sample sizes.

## Conclusions

Our investigation revealed a significant association between postoperative platelet levels and prognosis, suggesting its potential as a prognostic indicator following valve replacement in the elderly. Those with higher postoperative platelet levels had a better overall postoperative prognosis than those with lower levels. However, the necessity for further prospective randomized controlled studies with large sample sizes remains imperative.

## Data Availability

The datasets used and/or analyzed during the current study available from the corresponding author on reasonable request.

## Declarations

### Footnote

Reporting Checklist: The authors have completed the STROBE reporting checklist.
